# Phase I and pharmacokinetic study of irinotecan in combination with R115777, a farnesyl protein transferase inhibitor

**DOI:** 10.1038/sj.bjc.6601732

**Published:** 2004-03-23

**Authors:** A Sparreboom, D F S Kehrer, R H J Mathijssen, R Xie, M J A de Jonge, P de Bruijn, A S T Planting, F A L M Eskens, C Verheij, G de Heus, A Klaren, S Zhang, T Verhaeghe, P A Palmer, J Verweij

**Affiliations:** 1 Department of Medical Oncology, Erasmus MC – Daniel den Hoed Cancer Center, 3075 EA, Rotterdam, the Netherlands; 2 Department of Pharmaceutical Biosciences, Uppsala University, SE-751 24 Uppsala, Sweden; 3 Johnson & Johnson Pharmaceutical Research and Development, Beerse, Belgium

**Keywords:** irinotecan (CPT-11), R115777 (tipifarnib), phase I, farnesyl protein transferase inhibitor, pharmacokinetics

## Abstract

The aims of this study were to determine the maximum-tolerated dose (MTD), toxicity profile, and pharmacokinetics of irinotecan given with oral R115777 (tipifarnib), a farnesyl protein transferase inhibitor. Patients were treated with escalating doses of irinotecan with interval-modulated dosing of R115777 (continuously or on days 1–14, and repeated every 21 days). In total, 35 patients were entered onto the trial for a median duration of treatment of 43 days (range, 5–224 days). Neutropenia and thrombocytopenia were the dose-limiting toxicities; other side effects were mostly mild. The MTD was established at R115777 300 mg b.i.d. for 14 consecutive days with irinotecan 350 mg m^−2^ given every 3 weeks starting on day 1. Three patients had a partial response and 14 had stable disease. In the continuous schedule, the area under the curves of irinotecan and its active metabolite SN-38 were 20.0% (*P*=0.004) and 38.0% (*P*<0.001) increased by R115777, respectively. Intermittent dosing of R115777 at a dose of 300 mg b.i.d. for 14 days every 3 weeks is the recommended dose of R115777 in combination with the recommended single-agent irinotecan dose of 350 mg m^−2^.

Over the past decade, the development of various new technologies like genomics, high-throughput screening and combinatorial chemistry has resulted in an explosion in the number of potential targets for anticancer drugs (Anzick and Trent, 2002; McLeod, 2002). In addition, an improved understanding of signal transduction pathways has led to the identification of various G-proteins, including Ras, which are critical intermediates of cell signalling and cytoskeletal organisation (Adjei, 2001). Membrane localisation of Ras proteins is catalysed by the enzyme farnesyl protein transferase (FPT) and involves the addition of a farnesyl group to conserved amino-acid residues at the carboxyl terminus (Kato *et al*, 1992). This process brings Ras into proximity to growth factor receptors and coupling proteins allowing for activation of a cascade of phosphorylation events through sequential activation of the PI3 kinase/AKT pathway, which is critical for cell survival, and the Raf/Mek/Erk kinase pathway, which has been implicated in cell proliferation (Haluska *et al*, 2002).

As farnesylation of Ras is required for its activity, a series of FPT inhibitors has been designed as potential anticancer agents to abrogate its function (Rowinsky *et al*, 1999; Johnston, 2001). Among numerous FPT inhibitors synthesised, two orally bioavailable agents, sarasar (formerly SCH66336) (Ganguly *et al*, 2001) and R115777 (tipifarnib, Zarnestra) (Venet *et al*, 2003), have advanced to Phase II/III clinical development. The latter agent is an orally bioavailable methyl-quinolone and belongs to the class of nonpeptidomimetic FPT inhibitors with a broad spectrum of preclinical antitumour activity (End *et al*, 2001; Kelland *et al*, 2001; Smith *et al*, 2002). Phase I clinical trials with single-agent R115777 have been completed using both intermittent and continuous dosing regimens (Hudes *et al*, 1999; Zujewski *et al*, 2000; Karp *et al*, 2001; Punt *et al*, 2001; Crul *et al*, 2002). The most prominent dose-limiting side effects on regimens with twice-daily (b.i.d.) dosing for up to 21 days relate to myelosuppression. With continuous dosing (i.e., without rest periods), dose-limiting myelosuppression and peripheral neuropathy were seen. In these Phase I trials, evidence of activity was observed in a variety of solid tumours, including colon (Zujewski *et al*, 2000) and non-small-cell lung cancer (Crul *et al*, 2002). This observation provided the rationale for initiation of a series of Phase II and Phase III trials in breast (Johnston *et al*, 2002), colorectal (Cunningham *et al*, 2002), glioma (Cloughesy *et al*, 2002), non-small-cell lung (Adjei *et al*, 2002), pancreatic (Cohen *et al*, 2002; Macdonald *et al*, 2002; Van Cutsem *et al*, 2002), prostate (Haas *et al*, 2002), and small-cell lung cancer (Heymach *et al*, 2002). Activity has been noted in breast cancer (Johnston *et al*, 2002), in acute myelogenous leukaemia (Cortes *et al*, 2003) and myeloproliferative disorders (Gotlib *et al*, 2002). The absence of activity in gastrointestinal malignancies suggests that other directions for the development of this drug should be appraised. One of the most promising of these is the evaluation of combination regimens with classical cytotoxic agents with a distinctly different mode of action (Moasser *et al*, 1998).

Against this background, we initiated a Phase I dose-escalation trial to investigate the feasibility of the combination of R115777 given orally on a continuous or intermittent schedule and the topoisomerase I inhibitor irinotecan, a prodrug of SN-38, given intravenously once every 3 weeks. The objectives of this study were (i) to assess the safety and toxicity profiles of this combination; (ii) to determine the dose-limiting toxicities (DLTs), the maximum tolerable doses (MTDs), and the recommended doses for further trials; and (iii) to examine the effect of irinotecan on R115777 pharmacokinetics and *vice versa*.

## PATIENTS AND METHODS

### Eligibility criteria

Patients with a histologically or cytologically confirmed diagnosis of a solid malignancy refractory to standard therapy or for whom other treatment options were not available (e.g., pancreatic cancer), were eligible for the present study. Additional eligibility criteria included: (i) age at least 18 years; (ii) Eastern Cooperative Oncology Group performance status ⩽1; (iii) no previous treatment with antineoplastic agents for at least 4 weeks (or 6 weeks in case of nitrosoureas or mitomycin C), and no more than one prior chemotherapy regimen for advanced disease; (iv) no prior treatment with topoisomerase I inhibitors; (v) no known diagnosis of Gilbert's syndrome or any other important contraindication for treatment with the normal prescribed dose of irinotecan; (vi) no prior extensive (>25%) radiotherapy of the bone marrow region; and (vii) adequate hematopoietic (WBC count, >3.5 × 10^9^ l^−1^, and platelet count, >100 × 10^9^ l^−1^), renal (serum creatinine concentration, ⩽1.5 × upper limit of institutional normal (ULN)), and hepatic function (total serum bilirubin, ⩽1.5 × ULN; aspartate aminotransferase and alanine aminotransferase, ⩽2.5 × ULN or ⩽5 ULN in case of liver metastases). The study protocol was approved by the Erasmus MC Review Board, and all patients signed informed consent before study entry.

### Drug administration

Irinotecan (Aventis, Antony Cedex, France) was administered once every 3 weeks as a 90-min intravenous infusion after dilution of the pharmaceutical preparation in 250 ml of isotonic sodium chloride, with the drug dose normalised to a patient's body-surface area. Premedication consisted of ondansetron (8 mg intravenously) and dexamethasone (10 mg intravenously), both administered 30 min before irinotecan. Atropine (0.25 mg subcutaneously) was administered as a prophylaxis for irinotecan-induced acute cholinergic syndrome in case the patient experienced this side effect in the previous cycle. For delayed-type diarrhoea, patients received loperamide (4 mg orally), followed by a loperamide dose of 2 mg administered every 2 h for up to 12 h after the last liquid stool, without exceeding a total of 48 h of treatment. If diarrhoea persisted for more than 24 h, patients received a 7-day prophylactic antibiotic course with ciprofloxacin. R115777 (Johnson & Johnson Pharmaceutical Research and Development, Beerse, Belgium) was provided as 100-, 200-, or 300-mg tablets, and was administered orally at intervals of 12 h with or immediately after a meal.

### Study design

The dose-escalation schemes for R115777 and irinotecan were defined before the start of the study. R115777 was started at 300 mg b.i.d. in the first patient, but based on emerging data from other studies, for subsequent patients the starting dose was reduced to 200 mg b.i.d., with an escalating scheme for irinotecan, starting at 200 mg m^−2^ and escalating steps of 50 mg m^−2^ up to the registered single-agent dose, 350 mg m^−2^. Once the full dose of irinotecan was reached, further escalation of R115777 would take place in steps of 100 mg b.i.d. Irinotecan was always given on day 1 of the first 3-week cycle and on day 1 of subsequent cycles. In the continuous regimen, R115777 was administered on days 3–21 of cycle 1, and then continuously thereafter, starting again on day 1 of subsequent cycles. In the intermittent regimen, R115777 was administered on days 3–14 of the first cycle, and then starting again on day 1 of subsequent cycles, but without administration on days 15–21.

Three patients were accrued at the starting dose level, and in the absence of DLT, another three patients were entered at the next dose level. For safety reasons, the next dose level was not opened until at least three patients were assessable for toxicity in the first cycle. In case only one patient developed DLT (see below), the dose level was expanded with additional patients to a total of six. In case DLT was reached in ⩾2 of three or ⩾2 of six patients, dose escalation was ceased. The MTD (recommended dose) was defined as one dose level below the level at which ⩾2 of six patients experienced DLT.

### Toxicity and response evaluation

Toxicity was assessed by the National Cancer Institute Version 2.0 common toxicity criteria on a scale graded 0–4 (Available: http://ctep.cancer.gov/reporti ng/ctc.html (accessed: February 27, 2004)). DLT was defined as one or more of the following events: (i) grade 4 haematological toxicity lasting for more than 7 days and/or associated with fever; (ii) any grade 3 or 4 nonhaematological toxicity with the exception of untreated nausea, vomiting, and/or alopecia; and/or (iii) an interruption of treatment for more than 3 weeks due to unresolved toxicity. Only events occurring during the first two cycles of treatment were taken into consideration in defining DLT. Tumours were assessed radiologically before patients were enrolled on the study, and after every even-numbered cycle. Response definitions were based on World Health Organisation criteria (Available: http://www.who.int/homepage/ (accessed: February 27, 2004)).

### Sample collection and analysis

Plasma samples were collected for measurement of irinotecan, SN-38, and R115777 concentrations. Irinotecan and SN-38 pharmacokinetics were assessed on day 1 of cycle 1 and day 1 of cycle 2. Plasma samples were collected for the measurement of R115777 concentrations on day 8 of cycle 1 (i.e. in the absence of irinotecan co-administration), and again on day 1 of cycle 2 (i.e., in the presence of irinotecan co-administration).

Blood samples for pharmacokinetic analysis were drawn from a vein in the arm opposite to that used for irinotecan infusion, and collected in 10-ml glass tubes containing lithium heparin as an anticoagulant during the first and second cycles. Samples for irinotecan pharmacokinetics were obtained before drug administration; at 1 h after start of infusion; at 5 min before the end of infusion; and at 30 min, and approximately 1, 1.5, 2.5, 5.5, 9.5, 22.5, and 46.5 h after the end of infusion. Samples for R115777 pharmacokinetics were obtained immediately prior to administration; and at approximately 1, 2, 3, 5, 8, and 12 (before the next dose) h after administration. Blood was immediately processed to plasma by centrifugation for 5 min at 3000 × **g** (4°C), and was then stored at −80°C until the time of analysis. Plasma samples were assayed for total drug forms (i.e. lactone plus carboxylate) of irinotecan and its metabolite SN-38, as well as for R115777 by reversed-phase high-performance liquid chromatography as reported in detail elsewhere (Sparreboom *et al*, 1998; Crul *et al*, 2002). Concentrations of the metabolites SN-38 glucuronide, APC (7-ethyl-10-[4-*N*-(5-aminopentanoic acid)-1-piperidino]-carbonyloxycamptothecin), and R115777 glucuronide in plasma were not measured because of limited sample supply that precluded the required additional analysis on the same material.

### Pharmacokinetic data analysis

Concentration–time data of irinotecan, SN-38, and R115777 were analysed by standard noncompartmental methods using the software package WinNonlin version 3.1 (Pharsight, Mountain View, CA, USA). The peak concentration and the time to peak concentration were the observed values. The AUC (AUC_12 h_ for R115777; AUC_48 h_ for irinotecan and SN-38) was calculated by trapezoidal summation. The terminal half-life was estimated by linear regression of the log-transformed data. Parameter predictions of the lactone and carboxylate forms of irinotecan and SN-38 were calculated by previously developed models (Xie *et al*, 2002). The considered parameters included clearance and AUC. The latter parameter was simulated for irinotecan and SN-38 from time 0 to 100 h after the start of infusion in each patient for a standard dose of 350 mg m^−2^. This data analysis was performed using the software package NONMEM version VI (SL Beal and LB Sheiner, San Francisco, CA, USA) with pooling of data from patients administered R115777 continuously and intermittently, which was carried out in order to increase the power of detecting any significant association in view of the small sample size.

### Statistical considerations

For the noncompartmental pharmacokinetic parameters, an analysis of variance was performed to generate appropriate estimates allowing for the calculation of 90% confidence intervals. A comparison between treatments (R115777 *vs* R115777+irinotecan, and irinotecan *vs* R115777+irinotecan) was made for the parameters peak concentration and AUC. A general linear model that included factors of patients, dose, and treatment was used. The mean treatment ratio (combination therapy *vs* monotherapy) and the associated 90% confidence intervals were calculated for log-transformed data using the mean square error from the analysis of variance, expressed as a percentage. The noncompartmental parameters from the continuous and intermittent regimens of R115777 were analysed separately. Probability values (two-sided) of less than 0.05 were regarded as statistically significant. All statistical calculations were performed using JMP version 3.2.6 (SAS Institute, Carey, NC, USA).

## RESULTS

### Patients and treatment

A total of 35 patients (19 men and 16 women) was enrolled onto the study between April 1999 and July 2001 (Table 1

**Table 1:** Patient demographics

**Characteristic**	**Value**
*Number entered*	35
Male	19
Female	16
	
Age (years)	52 (34–75)^a^
Weight (kg)	74 (39–114)^a^
	
*Ethnicity*	
Caucasian	34
Asian	1
	
*Primary site*	
Cervical	1
Colorectal	17
Oesophageal	4
Pancreatic	4
Papilla of vater	1
Small intestinal	1
Unknown	7
	
*Previous therapy*	
None	2
Chemotherapy	26
Radiotherapy	7

a Median with range in parentheses.

). The majority of patients had a diagnosis of colorectal cancer, and 26 had previously failed on chemotherapy. Seven patients had only received radiotherapy prior to the start of treatment, because other treatment options were considered not available per the standard Dutch treatment guidelines. Patients with metastatic colorectal cancer were treated with relatively low doses of irinotecan in the early phases of this study, because at that time the use of irinotecan was not yet considered the standard treatment option for this indication. The first four cohorts of patients were treated with continuous R115777 at 200 mg b.i.d. In all, 17 subjects were treated at this dose in combination with irinotecan at 200 mg m^−2^ (four patients, including the first patient who received R115777 at 300 mg b.i.d.), 250 mg m^−2^ (*n*=4), 300 mg m^−2^ (*n*=3) or 350 mg m^−2^ (*n*=6) in 21-day cycles. The observation of the occurrence of cumulative fatigue as well as a pharmacokinetic interaction between R115777 and SN-38 (see below) resulted in the decision to change the administration of R115777 from a continuous to an intermittent schedule. A total of 18 additional patients were treated at R115777 doses of 200 mg b.i.d., 300 mg b.i.d. or 400 mg b.i.d. (*n*=6 in each group) given intermittently (days 1–14 every 21 days) in combination with irinotecan at 350 mg m^−2^. The median number of cycles was 2 (range, 1–10 cycles), and the median duration of treatment was 43 days (range, 5–224 days). The median daily drug dose administered was close to the planned dose for both drugs in each treatment group.

### Toxicity profiles

During the entire course of treatment, all patients experienced one or more adverse events, and these were of grade 3 or 4 severity in 22 patients. The majority of grade 3 or 4 adverse events had already occurred in cycles 1 and 2 (Table 2

**Table 2:** Incidence of grade 3 or 4 drug-related toxicity in cycles 1 and 2^a^

	**Continuous**			**Intermittent**			
**Dose level**	**1**	**2**	**3**	**1**	**2**	**3**	**Total (%)**
R115777 dose (mg)	200	200	200	200	300	400	
Irinotecan dose (mg m^−2^)	<300	300	350	350	350	350	
No. of patients studied	8	3	6	6	6	6	35
No. with grade 3 or 4 AE	1^b^	2	3^b^	1	2^b^	4	13 (37.1)
							
*Haematological toxicity*							
Anaemia	—	—	—	—	—	1	1 (2.9)
Febrile neutropenia	1	—	1^b^	—	—	1	3 (8.6)
Leukocytopenia	1	—	—	—	—	—	1 (2.9)
Neutropenia	—	—	—	—	—	1	1 (2.9)
Thrombocytopenia	—	—	1	—	—	—	1 (2.9)
							
*Nonhaematological toxicity*							
Bacterial infection	—	—	—	1	—	1	2 (5.7)
Diarrhoea	—	—	1^b^	1	—b	1	3 (8.6)
Fatigue	—	1	—b	—	—b	1	2 (5.7)
Hypotension	—	—	1	—	—	—	1 (2.9)
Nausea	—	—	—	—	—	1	1 (2.9)
Rash	—	1	—	—	—	—	1 (2.9)
Sepsis	1	—	—	—	—	—	1 (2.9)
ALT increase	—	—	—	—	1^b^	—	1 (2.9)
Vomiting	—	—	—	—	1	1	2 (5.7)

a The classification of drug-related includes a possible, probable, or very likely relationship to R115777 and/or irinotecan treatment. Numbers indicate number of patients, unless stated otherwise.

b At least one (additional) patient experienced grade 3 or 4 drug-related side effect in a cycle beyond 1 and 2.

AE=adverse effect.

). Overall, 16 patients (45.7%) had drug-related, grade 3 or 4 adverse events. The most frequently reported nonhaematological drug-related events were diarrhoea (*n*=5), fatigue (*n*=4), vomiting (*n*=3), and nausea (*n*=2). The most frequently reported grade 3 or 4 haematological events leading to treatment intervention were neutropenia/febrile neutropenia (*n*=5), and thrombocytopenia (*n*=2). Three patients had systemic infections as a result of neutropenia, and 13 were withdrawn due to severe adverse events, mostly due to vomiting, nausea, and diarrhoea (*n*=3 each). One patient died during the study of a bowel perforation, which was not considered related to the study medication. For all cohorts, DLTs were observed in a total of seven patients in cycles 1 or 2. The main DLTs were related to neutropenia (*n*=4). In the continuous dosing regimen (200 mg b.i.d. R115777), DLT was recorded for one of eight patients administered <300 mg m^−2^ irinotecan (febrile neutropenia), one of three patients given 300 mg m^−2^ irinotecan (fatigue), and one of six patients in the cohort given 350 mg m^−2^ irinotecan (febrile neutropenia and thrombocytopenia). The MTD was not formally established with the continuous R115777 regimen, although irinotecan was administered at the full-recommended dose. However, in later courses seven of 17 patients developed severe fatigue necessitating treatment discontinuation in one of them. The long-term treatment with this schedule will be difficult and is not recommended for further study. In the intermittent regimen (all given irinotecan at 350 mg m^−2^), DLT was recorded in one of six patients at 200 mg b.i.d. R115777 (neutropenia with infection), one of six patients at 300 mg b.i.d. R115777 (grade 3 aspartate aminotransferase) and two of six patients at 400 mg b.i.d. R115777 (febrile neutropenia and thrombocytopenia in one, and nausea, fatigue and vomiting in the other). The MTD was established at 300 mg b.i.d. R115777 administered for 14 days in 21-day cycles with irinotecan at 350 mg m^−2^ given every 3 weeks. At this dose level, the combination therapy was tolerated remarkably well (Table 3

**Table 3:** Drug-related nonhaematological toxicity at MTD^a^

	**Cycle 1**		**Cycle 2**		**Cycle ⩾3**	
	**Gr 1/2**	**Gr 3/4**	**Gr 1/2**	**Gr 3/4**	**Gr 1/2**	**Gr 3/4**
Nausea	5	—	4	—	4	—
Vomiting	5	—	3	1^b^	3	—
Diarrhoea	5	—	6	—	3	1
Abdominal pain	2	—	—	—	1	—
Dizziness	2	—	—	—	1	—
Insomnia/somnolence	2	—	1	—	—	—
Malaise	1	—	—	—	1	—
Fatigue	3	—	4	—	1	1^b^
Rash	1	—	1	—	—	—
Weight decrease	—	—	1	—	2	—
Alopecia	2	—	1	—	2	—

a The classification of drug-related includes a possible, probable, or very likely relationship to R115777 and/or irinotecan treatment. Numbers indicate number of patients out of a total of 6 (5 for cycle ⩾3) treated with R115777 at 300 mg b.i.d. administered for 14 days in 21-day cycles with irinotecan at 350 mg m^−2^ given every 3 weeks.

b Represents the same patient.

Gr=grade.

).

### Antitumour activity

Of 35 patients, 30 were assessable for response as per the protocol guidelines, but the following was analysed on an intention to treat basis. Three (8.6%) patients achieved a partial response to therapy, one each with papilla of vater adenocarcinoma, unknown primary adenocarcinoma, and moderately differentiated squamous cell carcinoma of the cervix. In addition, 14 patients (40%) had stable disease, and 13 (37%) patients had progression of disease.

### Irinotecan disposition

**Figure 1:**
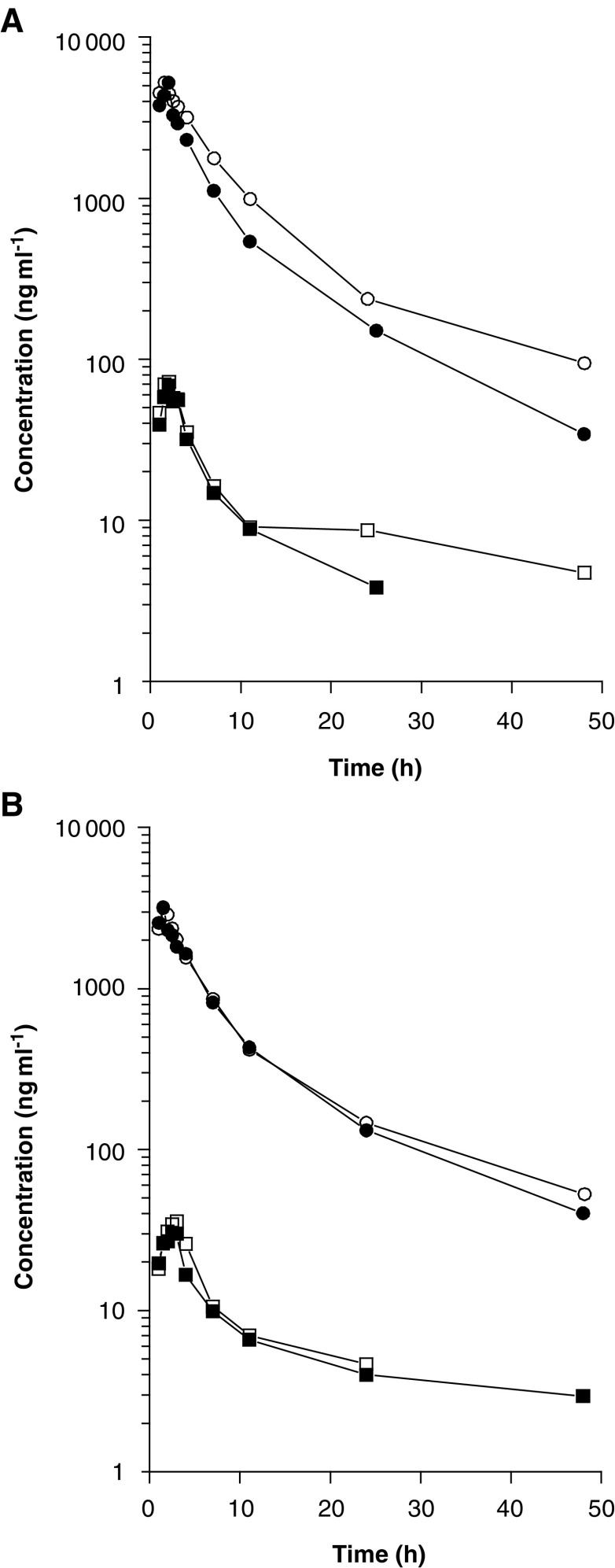
Plasma concentration *vs* time profiles of irinotecan (circles) and SN-38 (squares) in two representative patients treated with irinotecan at a dose of 350 mg m^−2^ as a 90-min infusion either given alone (closed symbols) or in combination with a 300-mg b.i.d. oral dose of R115777 (open symbols) given on a continuous (**A**) or intermittent (**B**) regimen.

The plasma concentration–time profiles of irinotecan and SN-38 were similar for all patients studied, with representative examples shown in Figure 1

. Over the various dose ranges studied, the AUC and the peak plasma concentrations of irinotecan increased in proportion with dose (*P*=0.43), albeit with substantial interpatient variability (i.e. greater than two-fold). In the absence of R115777, irinotecan and SN-38 pharmacokinetics, calculated by noncompartmental analysis, were very similar to previous single-agent data (Mathijssen *et al*, 2001). In the group of patients treated in combination with R115777 administered on a continuous schedule, the dose-normalised irinotecan AUC_48 h_ was 20.0% increased as compared to the control (*P*=0.004) (Table 4

**Table 4:** Noncompartmental analysis of irinotecan pharmacokinetics

**Parameter**	**(−) R115777**	**(+) R115777**	***P*-value**	**Ratio^a^**
*Continuous*^b^				
Irinotecan				
*C*_max_ (ng ml^−1^)	3889±801	3992±938	0.72	102 (92–113)
AUC_48 h_ (ng h ml^−1^)	20 891±3793	25 442±7011	0.004	120 (110–131)
*t*_1/2_ (h)	9.25±2.06	9.65±1.15	n/a	n/a
SN-38				
*C*_max_ (ng ml^−1^)	63.4±37.4	70.2±49.6	0.37	110 (92–132)
AUC_48 h_ (ng h ml^−1^)	397±195	569±328	<0.001	138 (123–156)
*t*_1/2_ (h)	13.4±4.44	21.4±9.76	n/a	n/a
				
*Intermittent*^c^				
Irinotecan				
*C*_max_ (ng ml^−1^)	3620±632	3710±620	0.54	103 (96–110)
AUC_48 h_ (ng h ml^−1^)	20 270±6020	22 580±6330	0.074	112 (101–124)
*t*_1/2_ (h)	10.1±1.00	10.0±1.30	n/a	n/a
SN-38				
*C*_max_ (ng ml^−1^)	41.5±20.1	42.9±19.7	0.55	104 (93–116)
AUC_48 h_ (ng h ml^−1^)	326±134	375±146	0.022	116 (105–128)
*t*_1/2_ (h)	18.1±13.1	19.3±10.3	n/a	n/a

*C*_max_=peak plasma concentration; AUC_48 h_=area under the plasma concentration–time curve up to 48 h after irinotecan administration; *t*_1/2_=half-life of the terminal disposition phase; *P*-value=probability value from a two-sided, paired Student's *t*-test; n/a=not applicable.

a Based on least-squares means calculated as the ratio of test (irinotecan with R115777) to reference (irinotecan alone), with 90% confidence limits (in log scale and expressed as a percent of single-agent irinotecan) in parentheses.

b Data were obtained from 11–13 patients receiving irinotecan (dose, 200–350 mg m^−2^) in the absence (cycle 1) or presence (cycle 2) of oral R115777 (dose, 200 mg b.i.d.) and analysed using noncompartmental analysis. Data are expressed as mean values±s.d., with *C*_max_ and AUC representing dose-adjusted values (to 350 mg m^−2^).

c Data were obtained from 13 patients receiving irinotecan (dose, 350 mg m^−2^) in the absence (cycle 1) or presence (cycle 2) of oral R115777 (dose, 200–400 mg b.i.d.), and analysed using noncompartmental analysis. Data are expressed as mean values±s.d.

). Likewise, the AUC_48 h_ of SN-38 was 38.0% increased (*P*<0.001) in the presence of R115777. After changing the R115777 administration to an intermittent schedule, the pharmacokinetic interaction was substantially less as a 12% (*P*=0.074) and 16.0% (*P*=0.022) increase in the AUC_48 h_ of irinotecan and SN-38, respectively, was observed (Table 4). Differences in irinotecan pharmacokinetics were also observed for the lactone and carboxylate forms, as estimated using a previously defined population model (Table 5

**Table 5:** Compartmental analysis of irinotecan pharmacokinetics^a^

**Parameter**	**(−) R115777**	**(+) R115777**	**Mean diff^b^**	***P*-value**
*Irinotecan*				
AUC_lac_ (ng h ml^−1^)	5380±727	5650±666	−269±101	0.067
AUC_car_ (ng h ml^−1^)	12 400±3710	14 300±4840	−1950±641	0.0027
CL_lac_ (l h^−1^)	80.6±16.6	73.1±16.6	7.44±2.35	0.998
CL_car_ (l h^−1^)	11.6±1.74	11.0±1.71	0.54±0.22	0.989
				
*SN-38*				
AUC_lac_ (ng h ml^−1^)	368±154	428±273	−59.7±34.7	0.049
AUC_car_ (ng h ml^−1^)	108±45.8	135±57.5	−26.7±10.1	0.0071
REC_lac_ (%)	6.91±3.05	7.48±4.45	−0.57±0.50	0.135
REC_total_ (%)	2.72±1.02	2.82±1.22	−0.09±0.13	0.227

**Figure 2:**
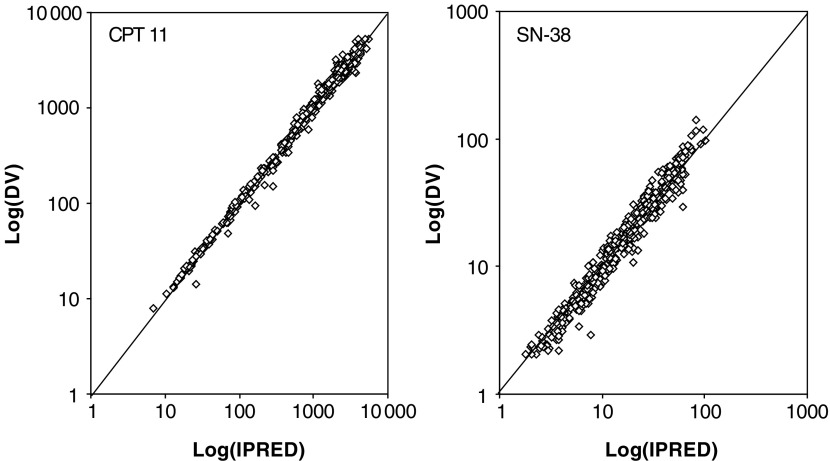
Logarithm of the individual predicted concentrations (Log IPRED) *vs* the observed concentrations (Log DV) of irinotecan (CPT-11; *left panel*) and SN-38 (*right panel*). All concentrations are in units of ng ml^−1^.

AUC=simulated area under the plasma concentration–time curve up to 100 h after drug administration; lac=lactone form; car=carboxylate form; CL=clearance; REC=relative extent of conversion (AUC_SN-38_/AUC_irinotecan_ × 100%); *P*-value=probability value from a two-sided, paired Student's *t*-test.

a Data were obtained from 26 patients receiving irinotecan (dose, 200–350 mg m^−2^) in the absence (cycle 1) and presence (cycle 2) of oral R115777 (dose, 200–400 mg b.i.d.), and analysed using a population pharmacokinetic model. Data are expressed as dose-normalised (to 350 mg m^−2^) mean values±s.d.

b Mean difference (cycle 2–cycle 1)±s.d.

). The observed plasma concentration–time profiles of irinotecan and SN-38 were well predicted by this model, as indicated by goodness-of-fit plots (Figure 2

).

### R115777 pharmacokinetics

The pharmacokinetic behaviour of R115777 was also very similar to previous single-agent data (Table 6

**Table 6:** Noncompartmental analysis of R115777 pharmacokinetics

**Parameter**	**(−) Irinotecan**	**(+) Irinotecan**	***P*-value**	**Ratio^a^**
*Continuous*^b^				
*t*_max_ (h)	2.0	2.1	n/a	n/a
*C*_max_ (ng ml^−1^)	840±551	735±377	0.75	93 (63–137)
AUC_12 h_ (ng h ml^−1^)	3240±1590	3000±1540	0.97	101 (68–149)
				
*Intermittent*^c^				
*t*_max_ (h)	2.0	2.0	n/a	n/a
*C*_max_ (ng ml^−1^)	610±248	663±296	0.55	106 (89–126)
AUC_12 h_ (ng h ml^−1^)	2820±1070	3170±1550	0.35	109 (93–127)

*t*_max_=time to peak concentration; *C*_max_=peak plasma concentration; AUC_12 h_=area under the plasma concentration–time curve up to 12 h after drug administration; *P*-value=probability value from a two-sided, paired Student's *t*-test; n/a=not applicable.

a Based on least-squares means calculated as the ratio of test (R115777 with irinotecan) to reference (R115777 alone), with 90% confidence limits (in log scale and expressed as a percent of single-agent R115777) in parentheses.

b Data were obtained from 11–13 patients receiving oral R115777 (dose, 200 mg b.i.d.) in the absence (cycle 2) or presence (cycle 1) of irinotecan (dose, 200–350 mg m^−2^), and analysed using noncompartmental analysis. Data are expressed as mean values±s.d., except *t*_max_ (median value).

c Data were obtained from 12–13 patients receiving oral R115777 (dose, 200–400 mg b.i.d.) in the absence (cycle 2) or presence (cycle 1) of irinotecan (dose, 350 mg m^−2^), and analysed using noncompartmental analysis. Data are expressed as dose-normalised (to 200 mg) mean values±s.d., except *t*_max_ (median value).

) (Zujewski *et al*, 2000; Crul *et al*, 2002). The peak plasma concentrations of R115777 were typically observed at 2 h after oral administration either with or without co-administration of irinotecan. On average, the increase in AUC (1.0%; *P*=0.97) and decrease in the peak concentration (7.0%; *P*=0.75) of R115777 in the combination therapy were not significant relative to those following continuous monotherapy. The substantial variability in both of these parameters for R115777 contributed to wide 90% confidence intervals (combination therapy *vs* R115777 alone). The slight increases in the AUC (9.0%; *P*=0.35) and peak concentration (6.0%; *P*=0.55) of R115777 in the intermittent regimens following administration of irinotecan were also not statistically significant. This suggests that irinotecan does not substantially influence the systemic disposition of R115777.

## DISCUSSION

This phase I study was performed to assess the safety and determine the MTD of a combination of twice-daily oral dosing of the FPT inhibitor R115777 and irinotecan administered in a once every 3 weeks schedule. Overall, the study demonstrates that this combination is fairly well tolerated, and that no unexpected toxicities were observed beyond those known with the respective single-agent regimens of both drugs. It was observed, however, that seven of 17 patients developed cumulative fatigue in the group of patients treated with continuous dosing of R115777. During the course of the trial, this observation combined with the notion of a pharmacokinetic interaction between R115777 and irinotecan prompted a change of R115777 dosage schedule from continuous to intermittent, consisting of drug dosing on 14 consecutive days every 21 days. In this intermittent regimen, DLT was observed at the standard dose of irinotecan of 350 mg m^−2^ and R115777 at a dose of 400 mg b.i.d., and consisted of febrile neutropenia in combination with thrombocytopenia or nausea, vomiting and fatigue. Other side effects were mostly mild and included rash and diarrhoea. The MTD was established at 300 mg b.i.d. of R115777 administered orally for 14 consecutive days in combination with irinotecan given at 350 mg m^−2^ every 3 weeks.

The pharmacokinetic data generated in this trial for irinotecan given alone were very similar to those described previously (Mathijssen *et al*, 2001). In the presence of R115777, however, the systemic exposure to total drug levels of irinotecan and its metabolite SN-38 was substantially increased, especially following continuous administration of R115777. Data from pharmacokinetic modelling further suggest that this interaction is most closely linked to effects on the carboxylate form of irinotecan. It was clearly beyond the scope of this investigation to unravel the mechanism behind the observed interaction. However, a possible explanation would be the fact that R115777 is known to inhibit CYP3A4 activity in human hepatic microsomal preparations, albeit at *in vitro* concentrations that are five-fold higher than the peak concentrations observed in the present study (Bohets, 1998). Previous investigations have shown that inhibition of CYP3A4 in patients on irinotecan treatment leads to shunting of parent drug to esterase-mediated hydrolysis to form SN-38 (Kehrer *et al*, 2002). The notion that the primary CYP3A4-mediated irinotecan metabolite APC is formed out of the carboxylate form of irinotecan (Xie *et al*, 2002), the pharmacokinetics of which are affected most, lends further support to a prominent role of CYP3A4 in the metabolism of irinotecan. However, evidence against inhibition of CYP3A4 activity by R115777 was observed in a previous interaction study with another CYP3A4 substrate, docetaxel; single-dose administration of R115777 (200 or 300 mg) was shown to have little effect on the systemic exposure to docetaxel. In a subset of subjects, the plasma AUC of docetaxel was relatively unchanged by continuous R115777 (200 mg b.i.d.) administration (unpublished data, Johnson & Johnson Pharmaceutical Research and Development).

Alternatively, as both irinotecan and R115777 are known to be extensively metabolised by UGT1A (Hanioka *et al*, 2001; Garner *et al*, 2002), it is also possible that competitive inhibition of this class of enzymes by R115777 results in impaired glucuronidation of SN-38, and hence leads to an increase in circulating levels of unconjugated SN-38 in plasma. However, recent preclinical studies suggest that UGT1A1, UGT1A7, and UGT1A9 are the major isozymes involved in SN-38 glucuronidation, with a minor role for UGT1A6, UGT1A8, and UGT1A10 (Gagne *et al*, 2002), whereas UGT1A4 is the prominent isozyme producing the *N*-glucuronide of R115777, with a minor role for UGT1A3 (Mannens *et al*, 2002). This makes an interaction between R115777 glucuronidation and SN-38 elimination at this level less likely.

Irinotecan undergoes complex and extensive biotransformation and elimination processes (Mathijssen *et al*, 2001). Besides oxidation and glucuronidation, irinotecan and SN-38 may be secreted by ABCB1 (P-glycoprotein), ABCC2 (MRP2 or cMOAT), and ABCG2 (BCRP or MXR). At present, it is unknown whether R115777 inhibits ABCC2 and/or ABCG2, or whether R115777 is a substrate for one or more of these transporters. There is some preliminary data for ABCB1 indicating that R115777 is not likely to be a substrate, and at physiologically relevant concentrations, R115777 does not significantly inhibit P-glycoprotein (unpublished data, Johnson & Johnson Pharmaceutical Research and Development). Therefore, the exact mechanism underlying the interaction between R115777 and irinotecan remains unclear and requires further investigation. However, the effect is apparently reversible, since a significant increase in irinotecan and SN-38 concentrations was observed following continuous R115777 administration but not following intermittent dosing. Most importantly, the clinical relevance of the observed interaction remains limited, since both irinotecan and R115777 could be safely given in combination at their full recommended single-agent doses.

The pharmacokinetics of R115777 given in the absence of irinotecan was also consistent with previous findings from patients on similar regimens (Zujewski *et al*, 2000; Crul *et al*, 2002). Recent data from a mass-balance study indicate that R115777 is very extensively metabolised to multiple products in addition to its major metabolite R115777-glucuronide (Garner *et al*, 2002). The major metabolic routes are a de-methylation on the quinolinone core as well as successive oxidation reactions of the C6-amino moiety resulting in loss of the methyl-imidazole moiety, which is most likely mediated by CYP3A isoforms. In an attempt to gain insight into the causes of the substantial interindividual variability in drug handling, an exploratory analysis of the associations between R115777 pharmacokinetics and genetic variants of genes with a putative role in its absorption and disposition characteristics is currently being performed.

In conclusion, intermittent dosing of R115777 at a dose of 300 mg b.i.d. for 14 consecutive days is feasible in combination with the standard dosage of irinotecan at 350 mg m^−2^ given once every 3 weeks.
